# Revelation of microcracks as tooth structural element by X-ray tomography and machine learning

**DOI:** 10.1038/s41598-022-27062-5

**Published:** 2022-12-28

**Authors:** Irma Dumbryte, Donatas Narbutis, Arturas Vailionis, Saulius Juodkazis, Mangirdas Malinauskas

**Affiliations:** 1 Institute of Odontology, Faculty of Medicine, Vilnius University, Vilnius, Lithuania; 2 Institute of Theoretical Physics and Astronomy, Faculty of Physics, Vilnius University, Vilnius, Lithuania; 3 Stanford Nano Shared Facilities, Stanford University, Stanford, USA; 4 Department of Physics, Kaunas University of Technology, Kaunas, Lithuania; 5 Optical Sciences Centre and ARC Training Centre in Surface Engineering for Advanced Materials (SEAM), School of Science, Swinburne University of Technology, Hawthorn, Australia; 6 WRH Program International Research Frontiers Initiative (IRFI) Tokyo Institute of Technology, Nagatsuta-cho, Midori-ku, Yokohama, Japan; 7 Laser Research Center, Faculty of Physics, Vilnius University, Vilnius, Lithuania

**Keywords:** Medical research, Optical techniques

## Abstract

Although teeth microcracks (MCs) have long been considered more of an aesthetic problem, their exact role in the structure of a tooth and impact on its functionality is still unknown. The aim of this study was to reveal the possibilities of an X-ray micro-computed tomography ($\mu$CT) in combination with convolutional neural network (CNN) assisted voxel classification and volume segmentation for three-dimensional (3D) qualitative analysis of tooth microstructure and verify this approach with four extracted human premolars. Samples were scanned using a $\mu$CT instrument (Xradia 520 Versa; ZEISS) and segmented with CNN to identify enamel, dentin, and cracks. A new CNN image segmentation model was trained based on “Multiclass semantic segmentation using DeepLabV3+” example and was implemented with “TensorFlow”. The technique which was used allowed 3D characterization of all MCs of a tooth, regardless of the volume of the tooth in which they begin and extend, and the evaluation of the arrangement of cracks and their structural features. The proposed method revealed an intricate star-shaped network of MCs covering most of the inner tooth, and the main crack planes in all samples were arranged radially in two almost perpendicular directions, suggesting that the cracks could be considered as a planar structure.

Microcracks (MCs), which can be clearly visible on the outer surface of the tooth, have long been considered more of an aesthetic problem for patients, but their exact role and impact on the integrity and longevity of a tooth is still unidentified (typical images of teeth with MCs are presented in Supplementary Fig. S1). To what extent patients’ concerns about the adverse effects of cracks on their teeth are justified, e.g. whether it is safe to bond brackets on cracked teeth, or whether dentists should take extra precautions when treating teeth with MCs—are questions that cannot be reasonably answered yet^1^. In the light of current knowledge, mainly based on lateral (two-dimensional, 2D) analysis of the tooth surface, these MCs usually should not cross the dentin-enamel junction (DEJ) and should not lead to any loss or visible separation of tooth structure^2^.

Although MCs are generally thought to be confined to the enamel, they are associated not only with the damaged appearance of the teeth, but also with a variety of undesirable and pathological consequences, such as compromised integrity of the enamel, effect on the sensitivity of the teeth, stain and plaque accumulation on the rough fractured surface, and an increased susceptibility to carious lesions^3–6^.

To date, the available information on teeth MCs and their characteristics has been obtained using 2D analysis techniques such as stereomicroscopy^7–10^, scanning electron microscopy (SEM)^1, 3, 11^ or three-dimensional (3D) scanning methods (optical coherence tomography (OCT) and ultrasound)^12–16^. The evaluation and measurement of qualitative and quantitative parameters of cracks (their number, direction, location, length, and width) presented in previously published literature describe the morphology and behaviour of MCs only on the outer enamel surface^1, 3, 7–9, 17–19^. Thus, it is still unknown whether these cracks are limited to the enamel or whether they can extend beyond the DEJ into the dentin or even the pulp.

In order to accurately assess the extent of possible damage (i.e. whether a MC crosses the DEJ and reaches the dentin or the pulp, what is its path throughout the tooth) to the underlying tooth structures in the area of MCs, it is necessary to perform their volumetric (3D) examination.

Several studies have been carried out so far on the depth parameter of cracks^13, 15, 20–22^. However, the limitations of the techniques used in those studies (e.g. the limited depth of penetration and scanning range of the device utilized for MCs analysis^15, 20^, sensitivity of the technique to surface curvature^13, 20^, the depth measurements carried out on a simulated human tooth^13^, an indirect method of determining the depth of the crack^21, 22^, the need for crack infiltration with contrast material for depth assessment^22^, or the physical measurement of the crack after cutting the tooth^21^) have all been the reasons behind the search and development of a new approach enclosing 3D imaging technique that would enable a non-destructive examination of MCs with micrometer resolution.

Advances in digital dentistry are followed by increasing attempts to computerize certain routine clinical procedures, particularly the analysis of radiographs^23, 24^. Artificial intelligence models for tooth and alveolar bone segmentation from cone-beam computed tomography images^23^, classification of cervical maturation degree and pubertal growth spurts from lateral cephalometric radiographs^24^ would reduce the need for manual evaluation of radiographic images and contribute to treatment efficiency. However, to accurately visualize the tooth structure, a higher resolution 3D imaging technique than the one which has been used so far is necessary.

The introduction of an X-ray micro-computed tomography ($\mu$CT) in dental studies has opened new possibilities for the measuring of enamel thickness and teeth, caries research, characterization of enamel white spot lesions and cortical bone microdamage, analysis of root canal morphology and preparation, detection of various types of teeth fractures, and dental tissue engineering^25–34^. This is an accurate 3D imaging technique that utilizes X-rays to see inside an object, not limited to slice-by-slice views^34^. One of the most important advantages of the method is the ability to provide volumetric information about the microstructure in a non-destructive way (today’s most advanced laboratory-based $\mu$CTs can achieve resolutions up to 0.7–1 ${\upmu \hbox {m}}$ (4–10 ${\upmu \hbox {m}}$ resolution is usually selected for biological samples) using geometrical magnification), and generally eliminating the step of an extensive sample preparation^34, 35^. In our recently published study X-ray $\mu$CT was validated as a method suitable for the 3D non-destructive visualization of enamel MCs with distinct precision and versatility^34^. However, it is assumed that the potential of this technique is much broader and that the proposed approach is fully expandable towards the more detailed teeth microstructure analysis^34^.

The aim of this in vitro study was to reveal the possibilities of an X-ray $\mu$CT in combination with convolutional neural network (CNN) assisted voxel classification and volume segmentation for 3D qualitative analysis of tooth microstructure and verify this approach with four extracted human teeth. Network of MCs had a typical cross sectional width (a local normal to the plane of crack) from $\sim$0.3–30 ${\upmu \hbox {m}}$, which spans two orders of magnitude.

## Methods

### Samples and study design

**Figure 1:**
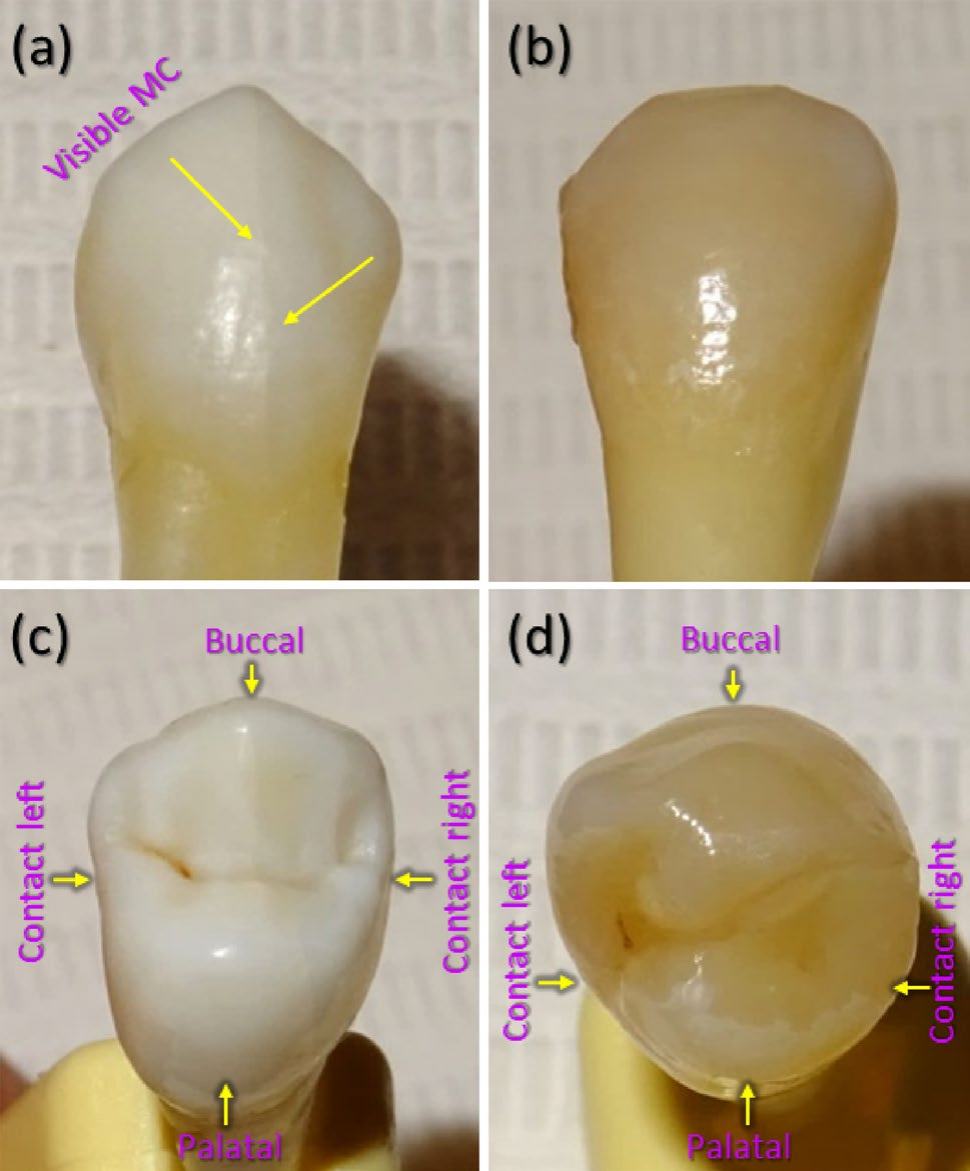
An example of photographs of two teeth with visible (**a**,**c**) and invisible (**b**,**d**) enamel microcracks (MCs) on the buccal tooth surface. Visible MCs and tooth surfaces are marked. Panels (**a**,**b**) show buccal view, while panels (**c**,**d**)—occusal view.

The study examined human maxillary premolars extracted for orthodontic reasons. The primary teeth selection criteria were as follows: (a) intact (i.e. healthy, undamaged) buccal, palatal, and contact surface enamel with no white spots, signs of dental fluorosis or enamel hypoplasia; (b) no pre-treatment with any chemical agents (such as hydrogen peroxide); (c) no previous orthodontic, endodontic or restorative treatment; (d) atraumatic tooth extraction procedure; (e) specimens correctly stored after extraction. The secondary criterion for the teeth selection was the presence of visible and invisible enamel MCs on the buccal enamel surface (Fig. 1)^1, 34, 36^. The current study did not require separate ethical approval for research on extracted teeth as it was carried out as part of a PhD project^1^ at Vilnius University (Lithuania), which has permission to use human samples for research purposes (No R-388 of 21 November 2008 “On approval of the provisions of the Vilnius regional biomedical research ethics committee”). Samples were collected, stored and used in accordance with the ethical guidelines for non-biomedical research involving human health (adapted from^37^). The teeth were prepared in accordance with the guidelines of the International Organization for Standardization (ISO/TS 11405; 2003)^38^, and were used with the patients’ informed consent and permission to utilize the obtained data for research purposes.

### Data acquisition

A more detailed description of the scanning procedure is available in a previously published study^34^. Here we provide a brief summary of experimental set-up. X-ray $\mu$CT scans with X-ray microscope (Xradia 520 Versa; ZEISS, Pleasanton, CA, USA) were used in the current study of a 3D distribution of cracks and other features in the teeth samples. The general layout of the experiment consisted of the X-ray source, the specimen, and the detector. The distances between the source-sample and sample-detector were adjusted to achieve the maximum magnification with the full field-of-view of the tooth sample. For this experiment the following distances were used: source-sample = ${21}\,\hbox {mm}$ and sample-detector = ${125}\,\hbox {mm}$. The sample projection images were obtained in absorption mode using geometrical magnification and a CCD detector resulting in a detector size of 2048 $\times$ 2048 pixels. To achieve the optimal signal-to-noise level (intensities of >5,000 grey value over low transmission regions), the exposure time of ${5}\,\hbox {s}$ was selected. The distances between the source, sample and detector resulted in a ${5\,\times 5\,\times 5\, {\upmu \hbox {m}}^{3}}$ voxel size which in this case defines the experimental resolution^34^. The final result of the scanning procedure was four data-cubes of $\sim$10$^3$ mm$^3$ ($\sim$ $2000^{3}$) voxels, containing values stored as 16-bit integers, with voxel edge of $\sim$ $5\,{\upmu \hbox {m}}$.

### Data preparation for analysis

**Figure 2:**
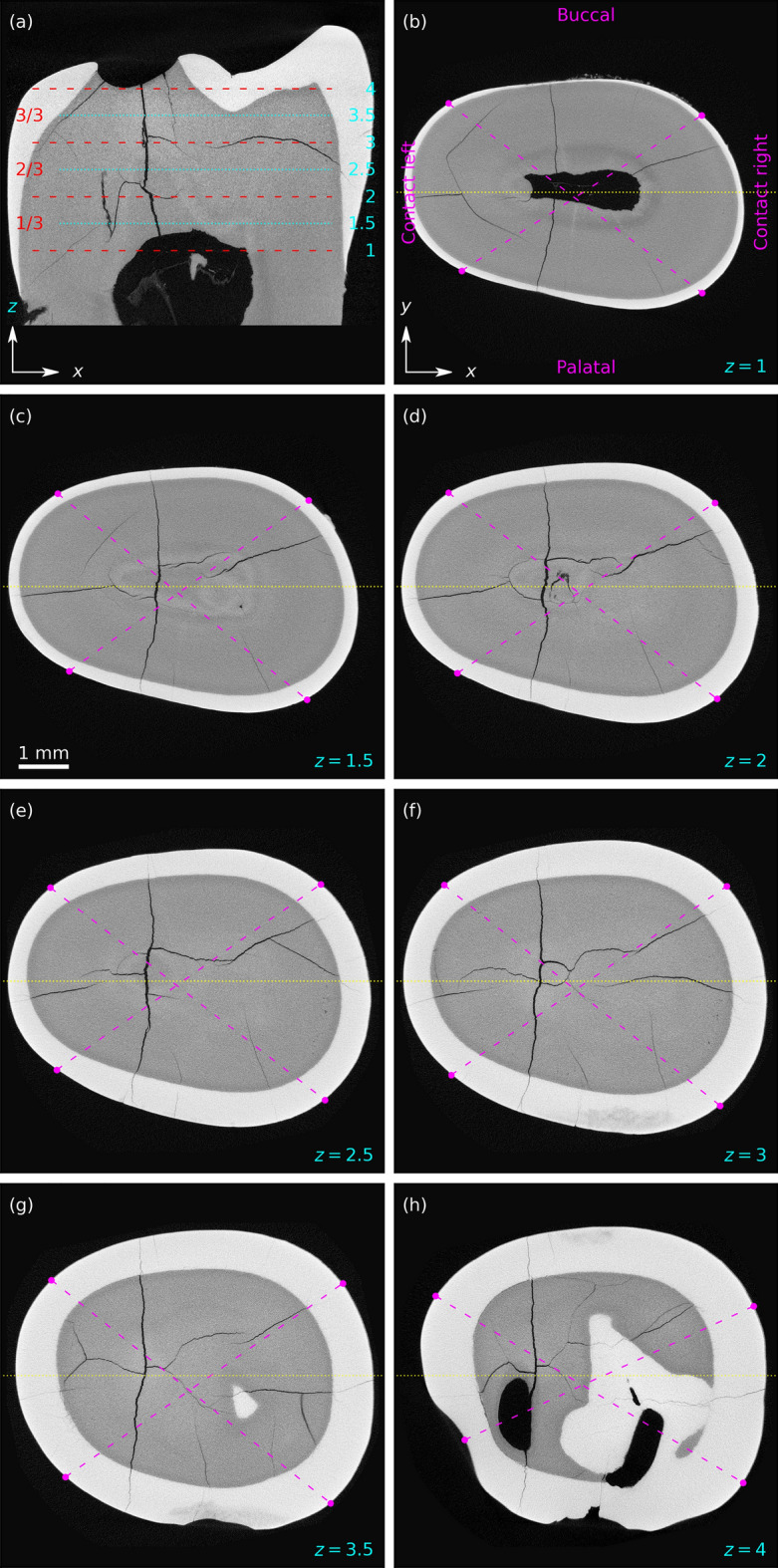
X-ray $\mu$CT data-cube cuts of a tooth *without visible* enamel microcracks on the buccal surface. Enamel is light-grey, dentin—dark-grey, and tooth outside, pulp and cracks—black. (**a**) Vertical slice in *x*, *z* plane at *y* position, as indicated by horizontal dotted yellow line in panels (**b**–**h**). (**a**) Horizontal dashed red and dotted cyan lines show positions $z=1 - 4$ of slices in *x*, *y* planes, displayed as images in panels (**b**–**h**). (**a**) Horizontal slabs selected between dashed red lines are indicated as 1/3, 2/3, 3/3. In panels (**b**–**h**) dashed magenta lines cut-out four identified sectors (buccal, contact right, palatal, contact left). Field of view is $\sim 8\times 8$ mm$^2$. One pixel corresponds to $\sim$10 ${\upmu \hbox {m}}$.

For faster visualization purposes, the data-cubes were resampled to a common voxel scale and aligned to principal contact surfaces of the teeth with $\sim$10 ${\upmu \hbox {m}}$ scale. In Fig. 2 axes *x*, *y*, *z* were aligned as indicated (*x* along the palatal, *y* along the contact, and *z* along the vertical extent of tooth). For detailed mapping of MCs, the vertical slice of the tooth was divided into three horizontal slabs of equal height corresponding to 1/3 (cervical third), 2/3 (middle third) and 3/3 (occlusal third) of the tooth surface. The division of the tooth surface was based on dental examination methodologies and different enamel quality and mechanical properties of the individual tooth (Fig. 2)^1, 4, 7, 36, 39–41^.

As each tooth has four surfaces of different convexity (Fig. 1), enamel thickness, distance to dentin and pulp, four sectors (buccal, contact right, palatal, contact left) were identified for the analysis (Fig. 2 (b–h))^42, 43^. Transitions from convex (buccal, palatal) to flatter tooth surfaces (contact right, contact left) were selected as reference points to define sectors. The dashed magenta lines connected the upper reference point of the right contact surface to the lower reference point of the left contact surface and vice versa. This resulted in $3 \times 4$ 3D slab sectors used for examination of MCs of each tooth. In each of the slab sector in Fig. 2, the following structures could be identified: enamel (visible as light-grey shaded area), dentin (dark-grey area surrounded by enamel), pulp (black area in the central part of the tooth), cracks (in the enamel, dentin, or both layers), scanning artefacts (large black cylinder, $\mu$CT playback “rays” on top of the tooth), enamel discoloration at the bottom.

Although the principal components of the tooth can be identified with a naked eye by the grey level of voxel value, such a straightforward selection of voxels to isolate tooth components would result in a rather poor quality, e.g. cracks, pulp, and outside of the tooth would have similar numerical values. Therefore, we trained a CNN to identify voxels (pixels in each slice since we processed data-cube as slices for this purpose) which belong to these four categories: (1) cracks, (2) enamel, (3) dentin, (4) air.

In the previous study^34^ a segmentation of a tooth was performed using deep learning toolkit within Dragonfly (Object Research Systems) software. It allowed to manually and iteratively derive robust eye-verified volume segmentation labels of a single tooth. In the current study this data was used to train a new CNN image segmentation model^44^, which was based on “Multiclass semantic segmentation using DeepLabV3+” example (by S. Rakshit, https://keras.io/examples/vision/deeplabv3_plus) and was implemented with “TensorFlow”^45^ (https://www.tensorflow.org). The model is a fully convolutional architecture with ResNet50 backbone^46^ (pretrained on ImageNet), encoder module that performs dilated convolutions as a multi-scale contextual information processor, and a decoder module that is responsible for accurate spatial boundary segmentation between categories.

As an input for training, slices of the tooth along *z* axis of 512 $\times$ 512 pixels were cropped and converted to RGB image repeating a 2D greyscale array three times, making it suitable for the ResNet50 input. As target labels, an array with the same spatial size, but with four channels (one-hot encoding the categories) was created, with any given pixel attributed to either cracks, enamel, dentin, or air. Categorical cross entropy loss function was used during training.

**Figure 3:**
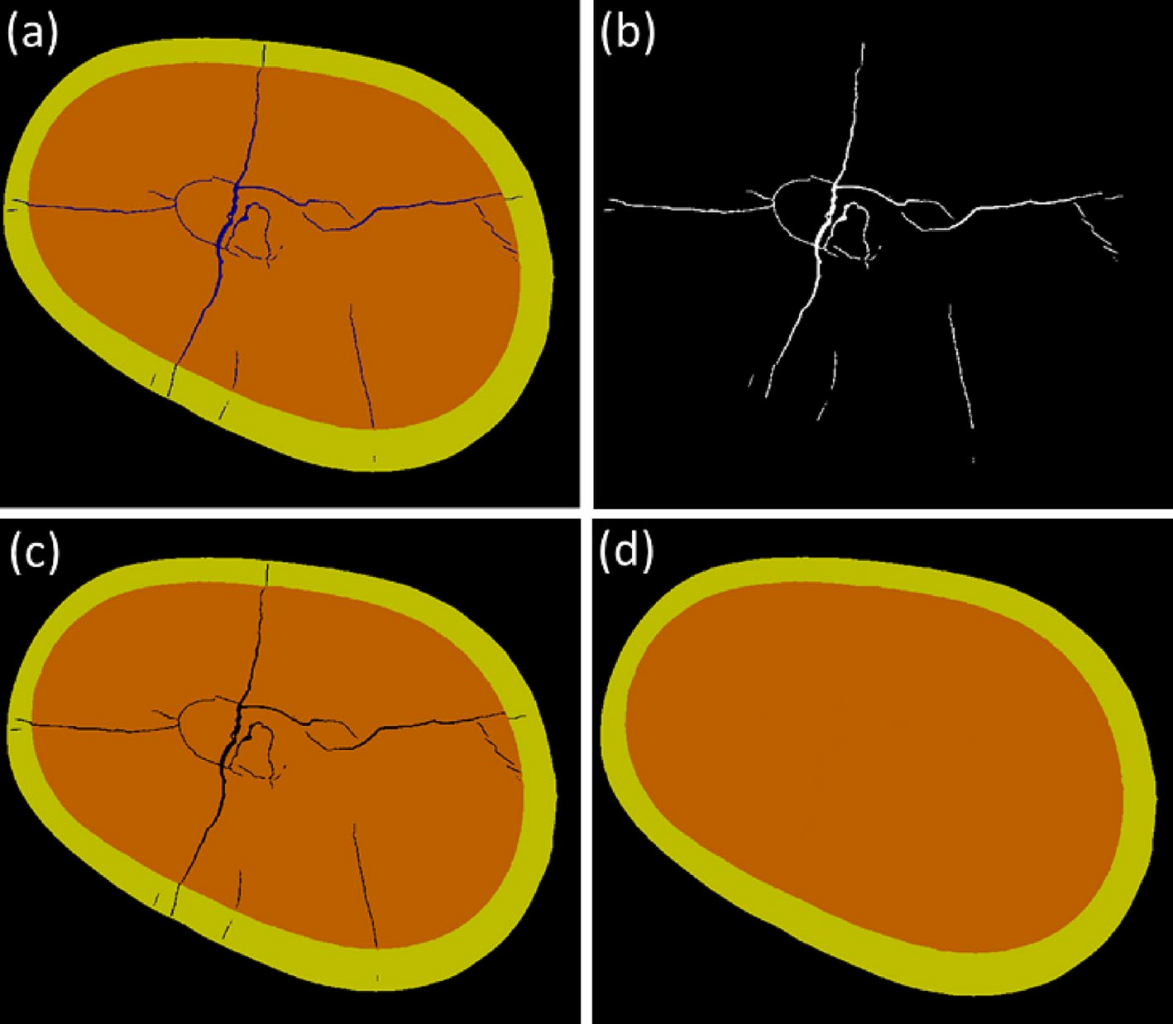
Example of X-ray $\mu$CT data-cube slice-by-slice segmentation with convolutional neural network (CNN) to produce data-cubes of structural elements of a tooth. (**a**) CNN’s predictions on slice image with enamel (yellow), dentin (brown), cracks (dark blue), and air (black) indicated. (**b**) Binary mask of cracks, which was used to fill in the segmentation image shown in (**c**), resulting in isolated areas of enamel and dentin presented in (**d**) as if there were no cracks in the tooth. One pixel corresponds to $\sim$10 ${\upmu \hbox {m}}$. “NumPy 1.24.0”^49^ (https://numpy.org) was used to process data-cubes, which were visualized with “Matplotlib 3.6.0”^50^ (https://matplotlib.org).

The model was trained for 50 epochs using Adam’s optimizer^47^, with a learning rate of $10^{-5}$, a batch size of 5 images, and random contrast and flip/rotation augmentations. It achieved $\sim$99.5% accuracy on both training and validation images (15,000 and 2,000 respectively) without over-fitting. An example of network’s predictions is presented in Fig. 3.

To process data-cubes of four teeth samples, each slice image ($\sim$1000 $\times$ 1000 pixels) was cropped into 512 $\times$ 512  pixel size overlapping tiles as suitable for CNN’s input, which yielded pixel classification maps of the same size that were assembled back to the size of the slice. The segmentation of each tooth data-cube was performed three times, taking slices perpendicular to *x*, *y*, and *z* axis. A voxel was identified as crack if it was classified as crack in at least two planes. The remaining voxels were classified as enamel, dentin, or air. A slice-by-slice fill-in was performed to restore enamel and dentin areas (as if the tooth had no cracks) using “scikit-image”^48^ (https://scikit-image.org) restoration.inpaint method (https://scikit-image.org/docs/stable/auto_examples/filters/plot_inpaint.html) on dilated mask of cracks.

Data-cube manipulations were performed using “NumPy 1.24.0”^49^ (https://numpy.org) while visualizations were created with “Matplotlib 3.6.0”^50^ (https://matplotlib.org) libraries. “SAOImageDS9” tool^51^ (https://sites.google.com/cfa.harvard.edu/saoimageds9) was used for visual assessment of iterative model training and the task of teeth segmentation. Although some minor segmentation artifacts can be noticed, the results of the teeth segmentation are robust (the CNN model architectures and training strategies are different between previous and this study), allowing to see the overall internal structure of teeth. The groups of connected cracks were isolated and filtered by size using “cc3d” library^52^ (https://github.com/seung-lab/connected-components-3d).

## Results

**Figure 4:**
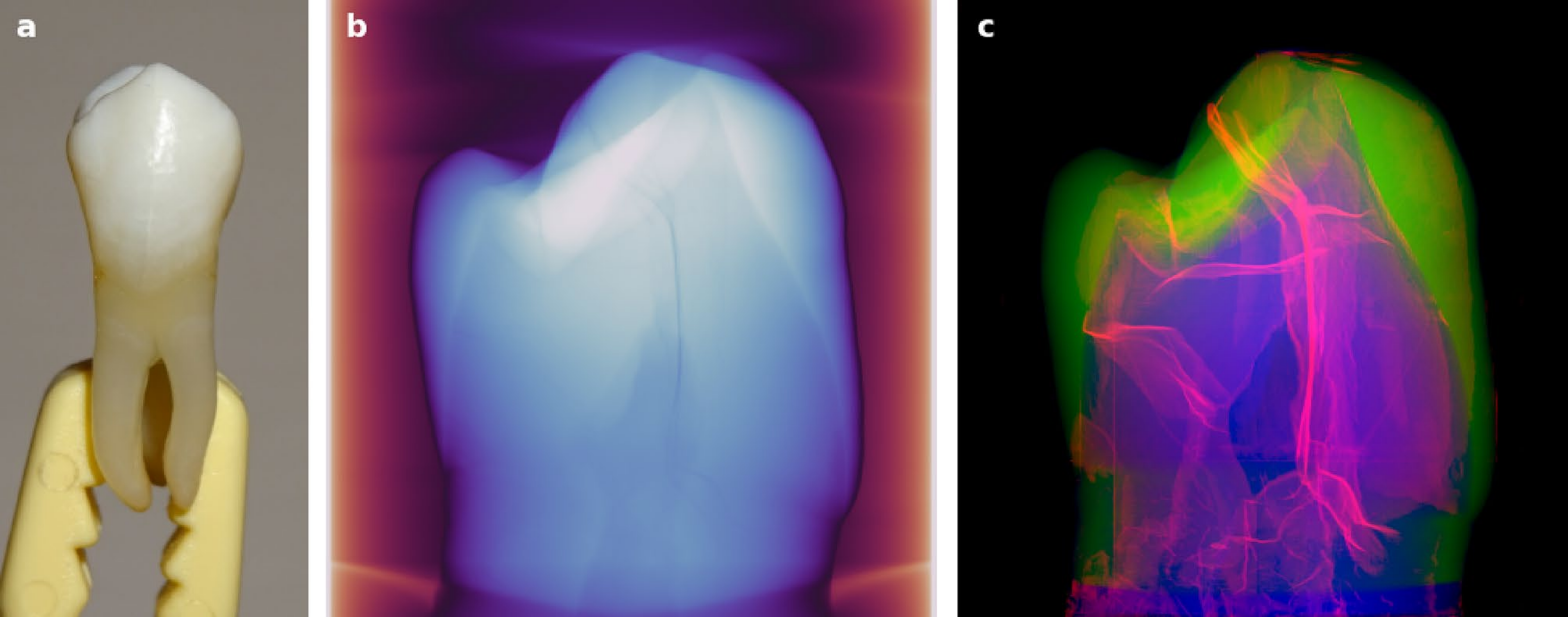
Example of a healthy human tooth extracted for orthodontic reasons (**a**). (**b**) X-ray $\mu$CT data-cube projected density map of the tooth with visible enamel, dentin, pulp, and cracks. (**c**) Voxel density projection of convolutional neural network segmented cracks, enamel, and dentin (red, green, blue) revealing intricate inner structure and suggesting that cracks could be considered an integral part of the tooth along with enamel, dentin, and pulp.

Four healthy undamaged human maxillary premolars (with and without visible enamel MCs on the outer tooth surface) that had been extracted for orthodontic reasons were analyzed using $\mu$CT together with CNN assisted segmentation. X-ray images of all the samples showed a dense tooth structure in which enamel, dentin, pulp, and cracks could be identified. The teeth appeared to be cracked, but without visible damage or separation of fragments. The network of cracks found in all the healthy teeth examined suggests that the cracks, along with the enamel, dentin, and pulp, could be considered a structural element of the tooth. The summary of the study is shown graphically in Fig. 4 with detailed results presented below.

### Characterization of microcracks

**Figure 5:**
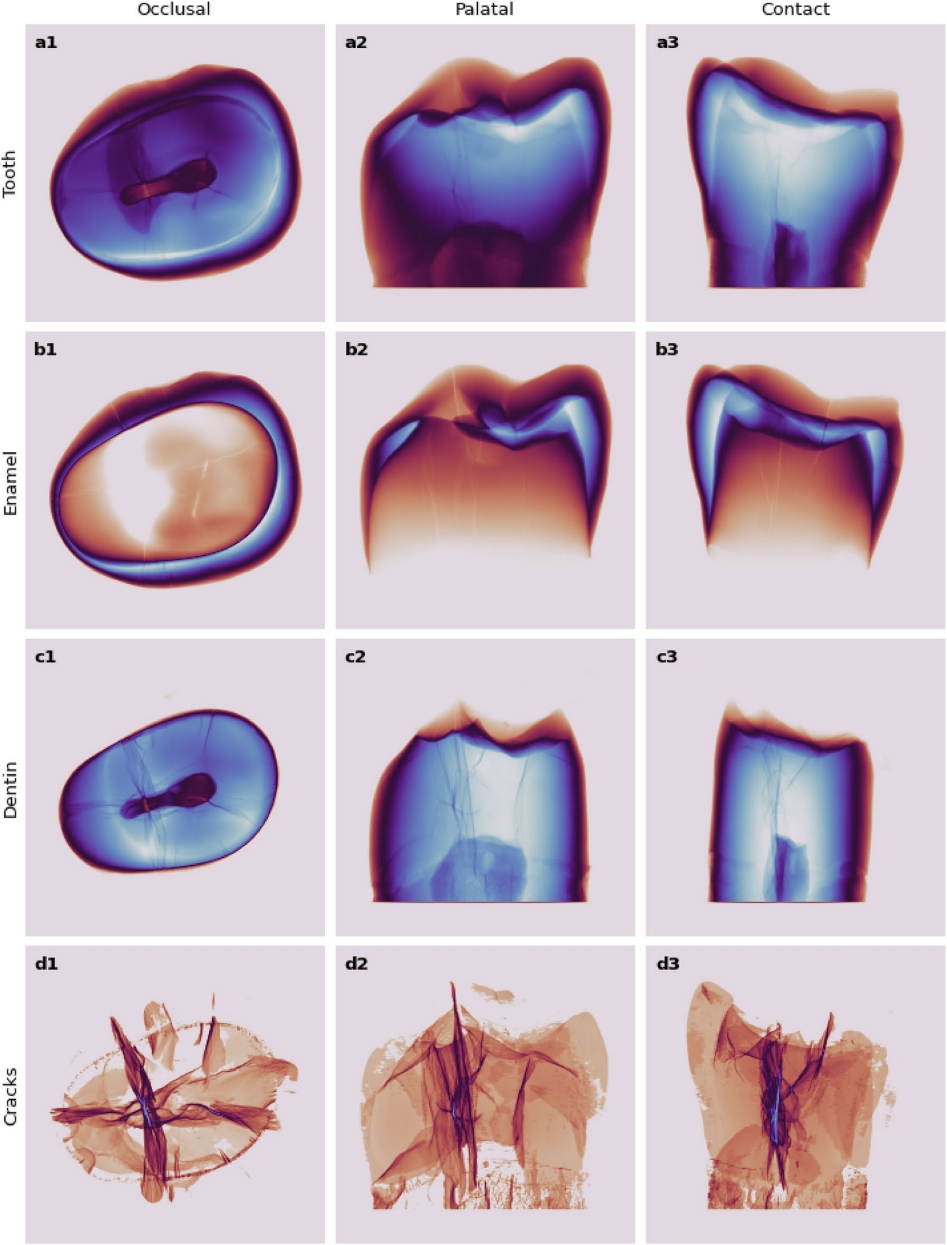
X-ray $\mu$CT data-cube projected density maps (bluish higher / brownish lower) along three major axes indicate convolutional neural network segmented voxels belonging to: (**a1**–**a3**) the tooth, (**b1**–**b3**) enamel, (**c1**–**c3**) dentin, and (**d1**–**d3**) cracks (color intensity scaled as square root for enhancement).

**Figure 6:**
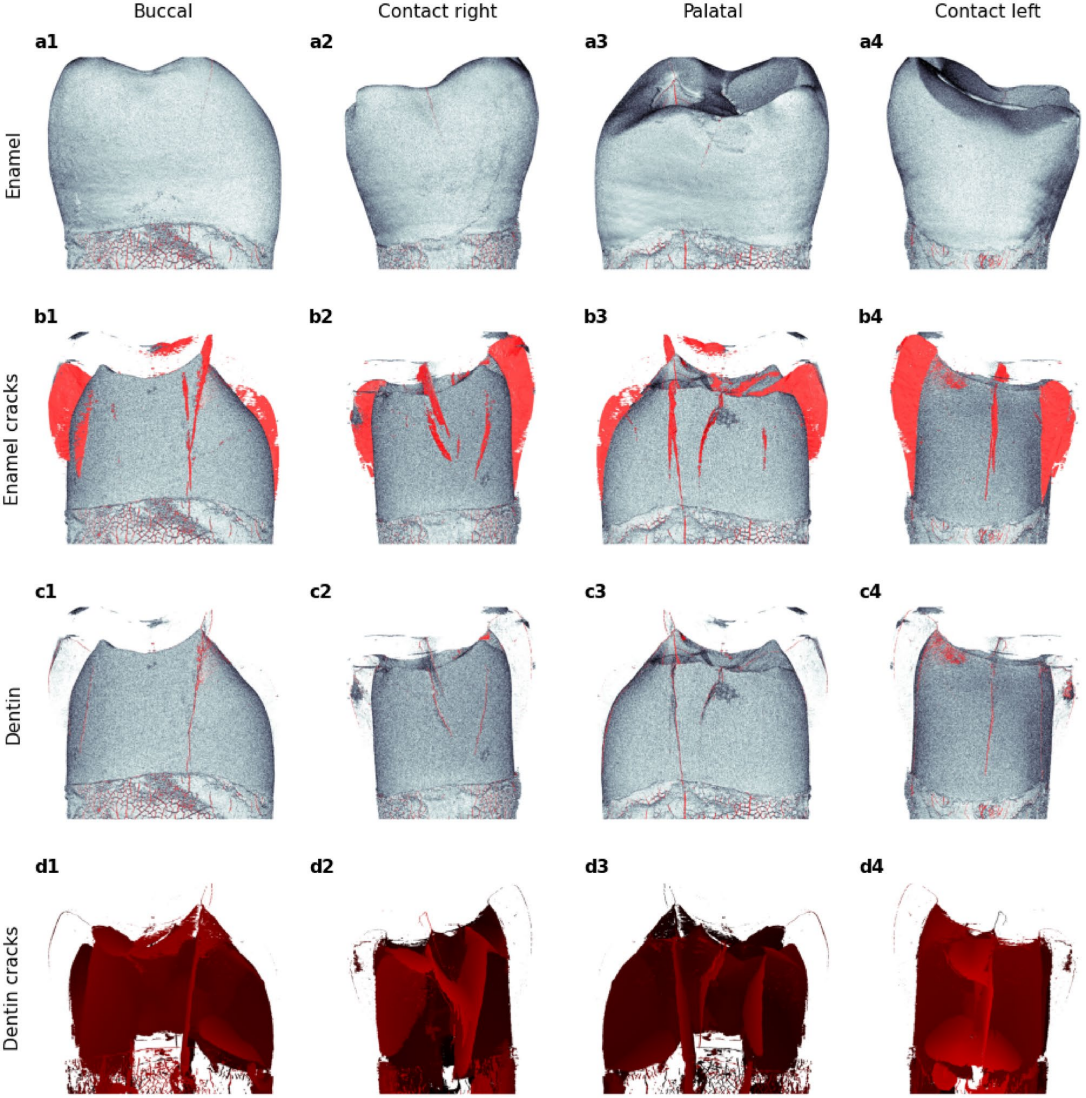
Views of four surfaces (buccal, contact right, palatal, and contact left) of the tooth. (**a1**–**a4**) Enamel surface and cracks (red), (**b1**–**b4**) with the enamel removed, dentin surface is visible with cracks within the enamel volume (red), (**c1**–**c4**) cracks on the dentin surface, and (**d1**–**d4**) cracks within the dentin volume shaded as distance along the line of sight (lighter red indicates distance closer / darker—further from the viewer).

X-ray $\mu$CT in combination with CNN assisted segmentation allowed to characterize all the MCs of a given tooth in three dimensions (Fig. 5, see Supplementary Movie 1). It was possible to clearly identify the MCs located on the buccal, palatal, and contact surfaces of the tooth and to determine in which volume of the tooth (e.g. enamel or dentin) they begin and extend (Fig. 6, see Supplementary Movie 2). The morphological characteristics of the different tooth surfaces, such as the degree of convexity, surface roughness, enamel layer width, did not interfere with the MCs assessment procedure. The fact that the MCs were visible on the outer surface or buried deep inside the tooth did not have any effect on their evaluation either.

### Evaluation of the arrangement of microcracks

**Figure 7:**
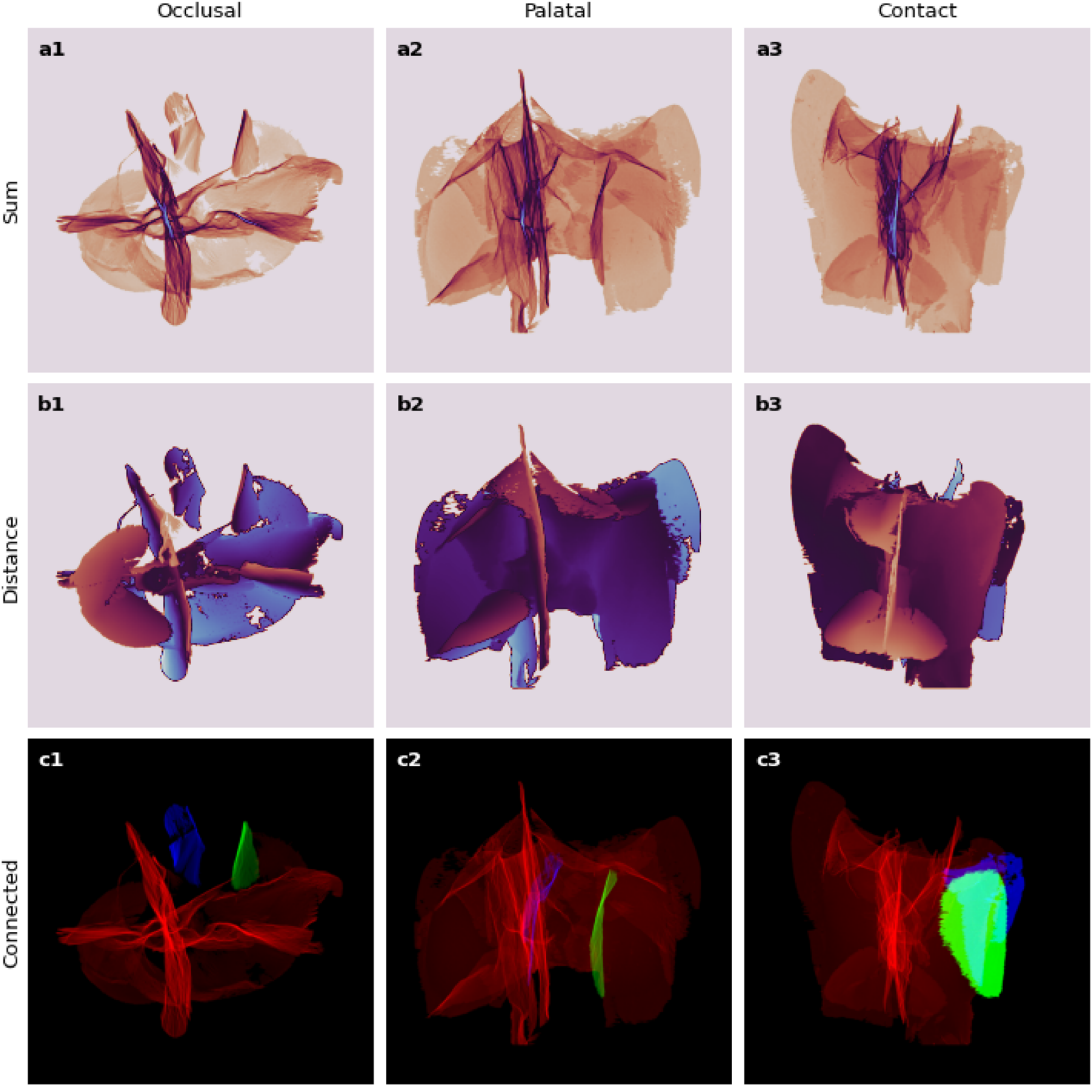
An intricate star-shaped network of cracks after removing small isolated groups. (**a1**–**a3**) Projected density maps (bluish represents higher / brownish lower). (**b1**–**b3**) Distance to the nearest crack voxel along the line of sight (bluish further / brownish closer). (**c1**–**c3**) Three largest connected crack groups isolated and colour-coded (red—largest, green—2nd largest, blue—3rd). “NumPy 1.24.0”^49^ (https://numpy.org) was used to process data-cubes, which were visualized with “Matplotlib 3.6.0”^50^ (https://matplotlib.org).

The applied scanning technique and developed segmentation approach allowed us to analyze the arrangement of MCs in all four teeth samples. Cracks that connect to each other have been identified and differentiated from those that are isolated. A single network of star-shaped cracks (longitudinally in relation to the main axis of the tooth) was found to cover most of the internal tooth structure (Fig. 7, see Supplementary Movie 3). This continuous connective formation occupies $\sim$2% of the volume of a tooth. In contrast, the remaining groups of single unconnected cracks tend to be located closer to the outer surface of the tooth and occupy a significantly smaller volume.

### Structural features of microcracks

**Figure 8:**
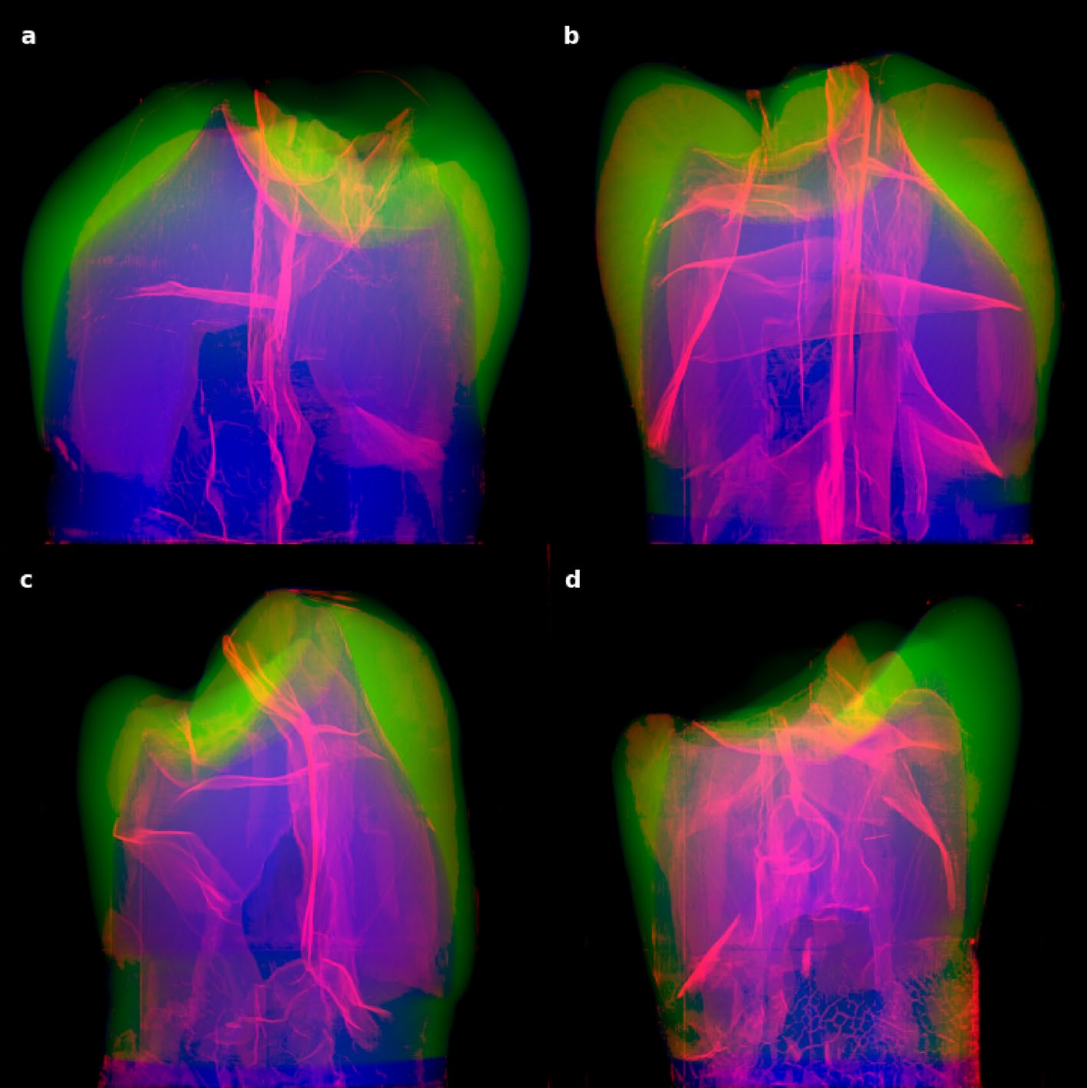
Four healthy teeth in our study sample (**a**–**d**) shown from random side views. X-ray $\mu$CT data-cube projected density maps of convolutional neural network segmented enamel (green), dentin (blue), and cracks (red, intensity scaled as square root for enhancement) reveal an intricate inner structure.

**Figure 9:**
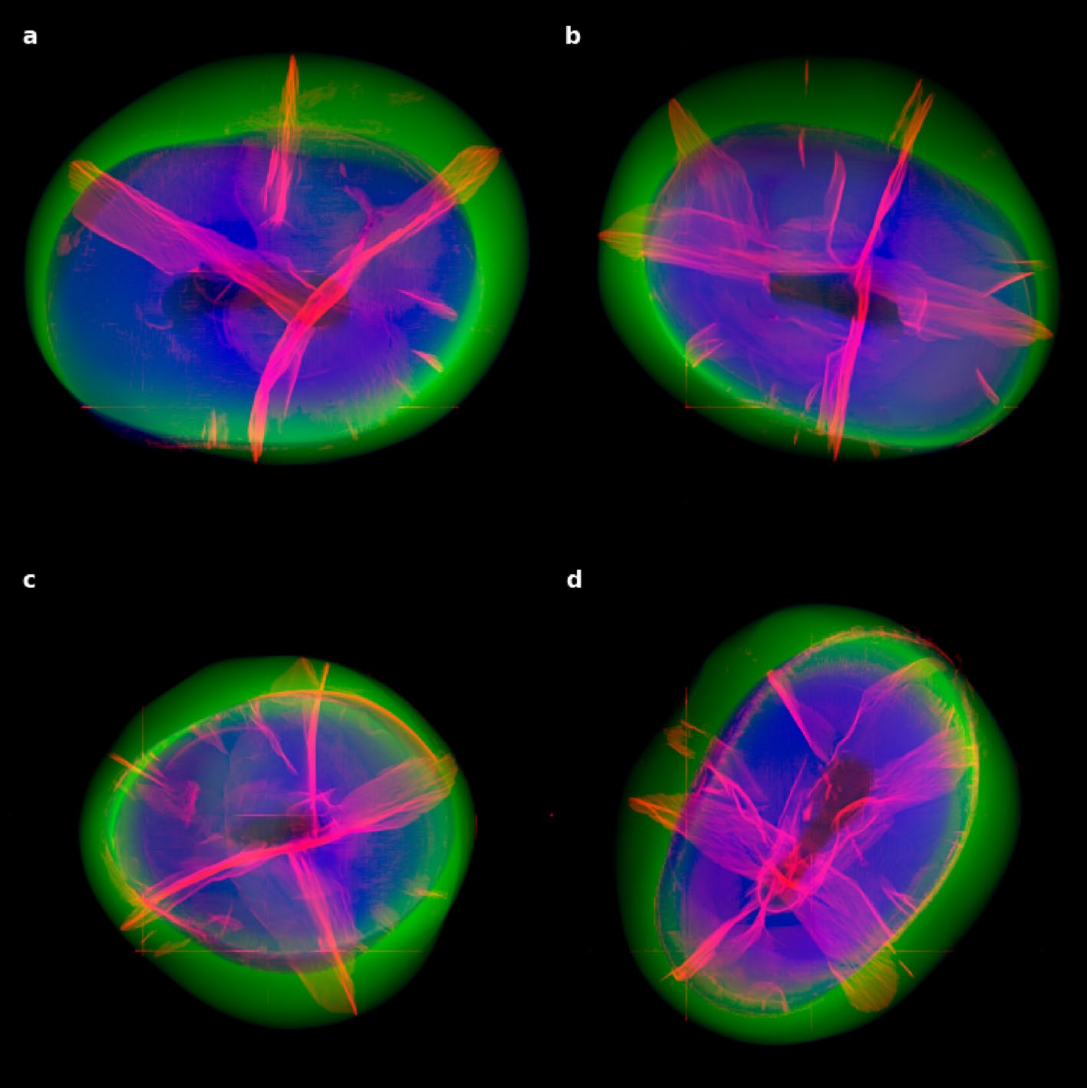
Same as Fig. 8, but the top (occlusal) view of our study sample of four healthy teeth. An intricate star-shaped network is visible.

The 3D visualization of the four teeth allowed us to evaluate the structural properties of MCs in each sample (Fig. 8, Fig. 9, and Supplementary Movie 4). In the microcrack network, it was possible to distinguish the main planes of the crack in two almost perpendicular directions, thus revealing the crack as a planar (interconnected manifolds) rather than a threaded structure (Fig. 7).

## Discussion

The current findings demonstrate that the proposed approach for the evaluation of healthy human teeth (scanning with X-ray $\mu$CT and CNN assisted segmentation) is capable of characterizing all MCs of a tooth regardless of the surface of the tooth in which they are located and of the layer of the tooth in which they begin and extend. Previously published studies^1, 13, 20, 21^ measured the following parameters of cracks: (a) length range, 0.24–10.15 ${\hbox {mm}}$^1^; (b) width range, 0.25–35.04 ${\upmu \hbox {m}}$^1^; (c) depth range, ${0.10\pm 0.01}\,\hbox {mm}$ of the craze lines^20^ and 0.8–1.0 $\hbox {mm}$ in the cracked tooth^20^; d) $\approx$ ${1.2}\,\hbox {mm}$ depth of the crack in simulated dentin^13^; e) 0.658–0.717 $\hbox {mm}$ crack depth calculated from the crack shadow^21^, and f) ${0.708}\,\hbox {mm}$ depth value physically measured by cutting the tooth^21^. Measurements of the quantitative crack characteristics have been performed using SEM^1^, laser ultrasonic system^20^, ultrasound^13^, indocyanine green near-infrared fluorescence^21^ and conventional near-infrared illumination^21^.

The proposed method is insensitive to the curved surfaces of the specimens used in the study, and as a result, it overcomes the shortcomings of the previous techniques of crack analysis^13, 20^. This is particularly important when the subject is a tooth having four surfaces of different convexity, of which the buccal surface is the most commonly examined and also the most convex. Compared to the 3D scanning methods used so far (OCT, ultrasound)^15, 20^, X-ray $\mu$CT allows the assessment of MCs at various distances from the outer enamel surface (enamel thickness $\approx$ ${0.5}\,\hbox {mm}$ in the cervical region, up to $\approx$ ${2.5}\,\hbox {mm}$ near the cusp for the molar teeth)^40^ or even in deeper layers of the tooth, e.g. the dentin, and is not affected by the different densities of these materials (density of enamel, ${2.61\pm 0.04}\, \hbox {g/cm}^{3} -- {2.77\pm 0.04}\, \hbox {g/cm}^{3}$, and dentin ${1.79\pm 0.02}\, \hbox {g/cm}^{3} -- {2.12\pm 0.03}\, \hbox {g/cm}^{3}$)^53^.

The aim of the study was to reveal the possibilities of the presented approach for 3D qualitative analysis of tooth microstructure on four intact (i.e. healthy, undamaged tooth with no previous orthodontic, endodontic or restorative treatment) human premolars. A single network of star-shaped cracks was identified to cover most of the internal tooth structure. The tendency of MCs to interconnect as they extend deeply from the outer enamel (the enamel closest to the tooth’s surface where the rods extend in a nearly parallel manner)^54^ to the inner tooth structures can be explained by the fracture-resistant properties of the enamel and dentin^55, 56^.

What has been known so far, is that the internal enamel (the enamel near the DEJ where the rods extend within groups that are obliquely oriented to one another) shows strong resistance to fracture and that crack growth resistance increases from outside to inside (fracture toughness of outer enamel at the tooth’s surface is ${0.67\pm 0.12}\,\hbox {MPa}\, \hbox {m}^{0.5}$, at inner enamel ${2.62\pm 1.39}\,\hbox {MPa}\,\hbox {m}^{0.5}/\hbox {mm}$)^40, 54, 55, 57, 58^. The rise in crack growth resistance within the inner enamel is explained by several mechanisms of toughening, including crack bridging, crack deflection, and microcracking (i.e. the ability of the enamel microstructure to promote guided growth and arrest of cracks)^34, 55^. Thus, it was expected to see that the MCs starting in the enamel would be stopped at the DEJ, which has unique biomechanical properties and provides a crack-arrest barrier for flaws formed in brittle enamel^56, 59^ by one of these fracture-resistant mechanisms. However, it should be noted that such phenomena will often be observed, but not in all teeth or in all cases.

Regarding the structure of the dentin, it has dentinal tubules surrounded by a thin mineral layer, which develop microscopic cracks under load^56^. Incipient cracks in dentin can propagate into a “sea of microcracks” that triggers a series of strong extrinsic mechanisms of toughening (fracture toughness of dentin is 1–2 $\hbox {MPa}\,\hbox {m}^{0.5}$ in directions perpendicular and parallel to the tubules)^56, 59^. The internal network of MCs revealed in our study justifies the term “sea of microcracks” mentioned above.

From the basics of mechanics, the stress concentration at the pre-existing crack depends on its shape and size as $\sigma _{\rm{max}} = 2\sigma \sqrt{l_{c} / l_{\rho }}$, where $2l_c$ is the length of the pre-existing crack (assumed elliptical) and $l_\rho$ is its curvature at the tip and $\sigma$ is the applied stress ^60^. The theoretical stress of crack formation is $\sigma _{\rm{th}} = \sqrt{\frac{\gamma E}{a_0}} \approx E/2\pi$ and propagation starts when $\sigma _{\text{max}}=\sigma _{\text{th}}$; *E* is the Young modulus, $\gamma$ is the surface energy per area and $a_0$ intra-atomic plane distance. The critical fracture toughness of dental enamel has orientation dependent anisotropy with perpendicular $K_{Ic}^\perp = 1.24$ MPa.$\sqrt{\textrm{m}}$ and in plane $K_{Ic}^\parallel = 0.70$ MPa.$\sqrt{\textrm{m}}$ values defined by orientation of hydroxyapatite nanocrystals ^61^. The brittle fast fracture was observed in the load-displacement tests for the perpendicular orientation and a slow continuous crack growth in in-plane (parallel) orientation. This anisotropy is related with sizes of nano-crystallites of 60-80 nm wide and $2~\mu$m long in the enamel. Cracks of comparable dimensions are expected at the initiation stage and define the required spatial resolution for imaging. The criterion of brittle fracture is $K\ge K_{Ic} = \sigma _c\sqrt{\pi l}$ for the *l* length of a pre-existing (linear) crack in a plate under tensile stress; for an elliptical crack it is by a fraction smaller $K_{Ic} = \frac{2}{\pi }\sigma _c\sqrt{\pi l}$ and $\sigma _c=\sqrt{\frac{2\gamma E}{\pi l}}$. An order of magnitude estimate of the crack propagation threshold stress for a $l = 2~\mu$m crack in enamel is $\sigma _c\equiv \frac{K_{Ic}^\perp }{\sqrt{\pi l}} \approx 4.9$ GPa. A maximum strength of a human bite of $\sim 100$ kg concentrated over an area of $1\times 1$ cm$^2$ (a single tooth) would correspond to pressure of only $10^7$ Pa or 0.2% of $\sigma _c$. The observed dental crack planes—preferentially along and perpendicular to the compressive stress experienced by a tooth—reduce propensity for crack propagation at smaller angles (along the direction of the tooth) as can be concluded from experiments with building materials subjected to compressive stress^62^.

It is important to emphasize that only healthy undamaged teeth (with or without visible MCs on the outer surface) were selected as the study sample. All the examined premolars were removed atraumatically (i.e. the root of the tooth was gently separated from the periodontal ligament using a special instrument; low pressure and constant force; no lateral, rotational or traction movements) by an experienced oral surgeon^63^. However, it has to be acknowledged that the experience of an oral surgeon reduces, but does not eliminate, the possibility of new MCs during extraction. Although the teeth were not loaded (i.e. not subjected to external forces during the study), the network of MCs was still clearly visible as a continuous connective element in all examined samples. This could reflect the structural nature of the internal network of MCs and lead to the hypothesis that it has a protective function (i.e. redistribution of forces).

In the mouth, teeth are subjected not only to masticatory forces or orthodontic stress during treatment with fixed appliances, but also to various parafunctions (e.g. bruxism), which can lead to occlusal overload and a higher risk of enamel damage^1^. There are two main groups of known tooth protection mechanisms that help withstand lifelong stress: (a) accommodative function of the periodontium (soft and hard tissues that surround the root of the tooth and change their anatomical and physiological features in response to occlusal forces)^64^, and (b) the structural and mechanical properties of the enamel (the most highly mineralized tissue of the human body, consisting of 96% mineral, 1% protein, and 3% water by weight)^34^. Enamel has all the characteristics of an anisotropic material, i.e. its mechanical properties such as hardness ($\sim$3–6 $\hbox {GPa}$), elastic modulus (70–120 $\hbox {GPa}$), and brittleness vary depending on the location, chemical components, and arrangement patterns of the enamel rods^1, 40, 65–67^, and thus contribute to the redistribution of the forces (guiding crack arrest, microcracking phenomenon)^34, 55^ and to the protection of the tooth’s internal structures against damage.

The results of this study show that cracks can still propagate through the DEJ into the dentin, so the DEJ could no longer be considered as an absolute barrier to arresting MCs. However, despite the fact that occlusal stresses can reach and affect not only the enamel but also the dentin, the tooth retains its structural integrity. Therefore, based on the results of the study, our hypothesis is that the continuous connective MC network found in the healthy tooth may be the missing link in order to answer the question of how the tooth is able to withstand the full range of occlusal forces without damage and fragmentation—*a third tooth protection mechanism* for the structural stability and adaptation to stress.

MCs can be confused with developmental defects of enamel (e.g. enamel lamellae). However, studies have shown that cracks and enamel lamellae are not identical^68^. Enamel lamellae can be described as fluid-filled cracks in the enamel (containing organic substances) that extend from the DEJ to the surface of the enamel, or vice versa^68, 69^. It has been observed that during the decalcification, the cracks disappear and the lamellae persist as a coherent organic, sheetlike process^68^. Meanwhile, the enamel cracks of the extracted tooth remain empty as no organic material is left^68, 69^. It should be noted that no new studies on lamellae have been carried out in the last decade. Therefore, in order to update the available information on enamel lamellae and to further elucidate the differences between cracks and lamellae, it is necessary to analyze enamel developmental defects using state-of-the-art techniques.

The available literature presents evidence of the correlation between the tissue dehydration and the dynamic dimensional changes within dentin and enamel^70^, as well as between dehydration and the fatigue crack growth resistance^71^. Due to the characteristics of the X-ray scanner used in this study^34^, the samples could not be stored in an aqueous media during the scanning procedure. Although it is not known exactly what effect the storage of the samples in non-hydrated media may have had on the cracks located in the crown of a tooth, it has already been shown that the width and length values of enamel MCs are not affected by the dehydration that occurs during the preparation of the samples for SEM scanning and observation (no new MCs were registered either)^1, 72^.

Our study had limitations. Firstly, the small sample size prevented us from drawing more general conclusions. However, by combining X-ray $\mu$CT together with machine learning, we have presented a novel method to analyze the 3D tooth microstructure and verified it using four extracted human teeth. Thus, we believe that despite the small number of specimens, the demonstrative value of our experimentally validated approach is high. In the future, we plan to test the method on a larger sample size, which would also increase the probative value of the work and its reliability as well as its versatility. Secondly, even after an atraumatic tooth extraction procedure, it cannot be guaranteed that cracks will not develop. It has to be acknowledged that the experience of an oral surgeon reduces, but does not eliminate, the possibility of new MCs. However, every effort was made to select only healthy, undamaged teeth as our study sample. Thirdly, due to the technical characteristics of the X-ray scanner, the specimens could not be stored in an aqueous media during the scanning procedure. Although it is not known exactly to what extent dehydration can affect teeth cracks, the inability to avoid it completely during the study could be considered as one of the limitations of our work. Finally, training the segmentation model is a subjective task, requiring visual decisions when selecting/rejecting groups of voxels and attributing labels to them, as there is no exact ground-truth, and the whole process is limited by scanning and reconstruction technique, noise, and error propagation, affecting classification power of the model, which could be increased once volume segmentation is performed with 3D convolution kernels instead of slice-by-slice 2D aggregate.

## Conclusions

The presented novel technique—using X-ray $\mu$CT in combination with CNN assisted segmentation - reveals the possibilities for a non-destructive and comprehensive 3D qualitative analysis of tooth microstructure. This method allows 3D characterization of all MCs of a tooth, regardless of the volume of the tooth in which they begin and extend, as well as the evaluation of the arrangement of cracks and their structural features. Anatomical characteristics of the tooth, such as enamel thickness, surface convexity or roughness, should no longer be a barrier to analyzing MCs with the described technique.

Using the proposed approach, a network of MCs inside all four healthy teeth (with or without visible MCs on the buccal surface) has been revealed, suggesting that the cracks could be considered as one of the structural and possibly functional (i.e. serving the function of redistribution of forces) elements of the tooth, with a protective, rather than a damaging function.

Detailed volumetric imaging of the MCs of a tooth expands our understanding of the cracking pattern in natural hard materials and allows us to gain more insight into how biologically inspired structures could be designed to predict the propagation of cracks in solid materials. From a clinical point of view, there is a need to revise the definition of MC that has been used so far, to re-evaluate the role and impact of these cracks on the integrity and longevity of the tooth, and to develop new algorithms for the monitoring and treatment of teeth with MCs in daily clinical practice.

## Supplementary Information

Supplementary Information 1.

Supplementary Information 2.

Supplementary Information 3.

Supplementary Information 4.

Supplementary Information 5.

Supplementary Information 6.

## Data Availability

All data generated or analyzed during this study are included in this published article [and its supplementary information files].
