# Supplementary figures and images for: Revelation of microcracks as tooth structural element by X-ray tomography and machine learning
Source: Sci Rep. 2022 Dec 28;12:22489. doi: 10.1038/s41598-022-27062-5 (PMC9797571; doi:10.1038/s41598-022-27062-5)

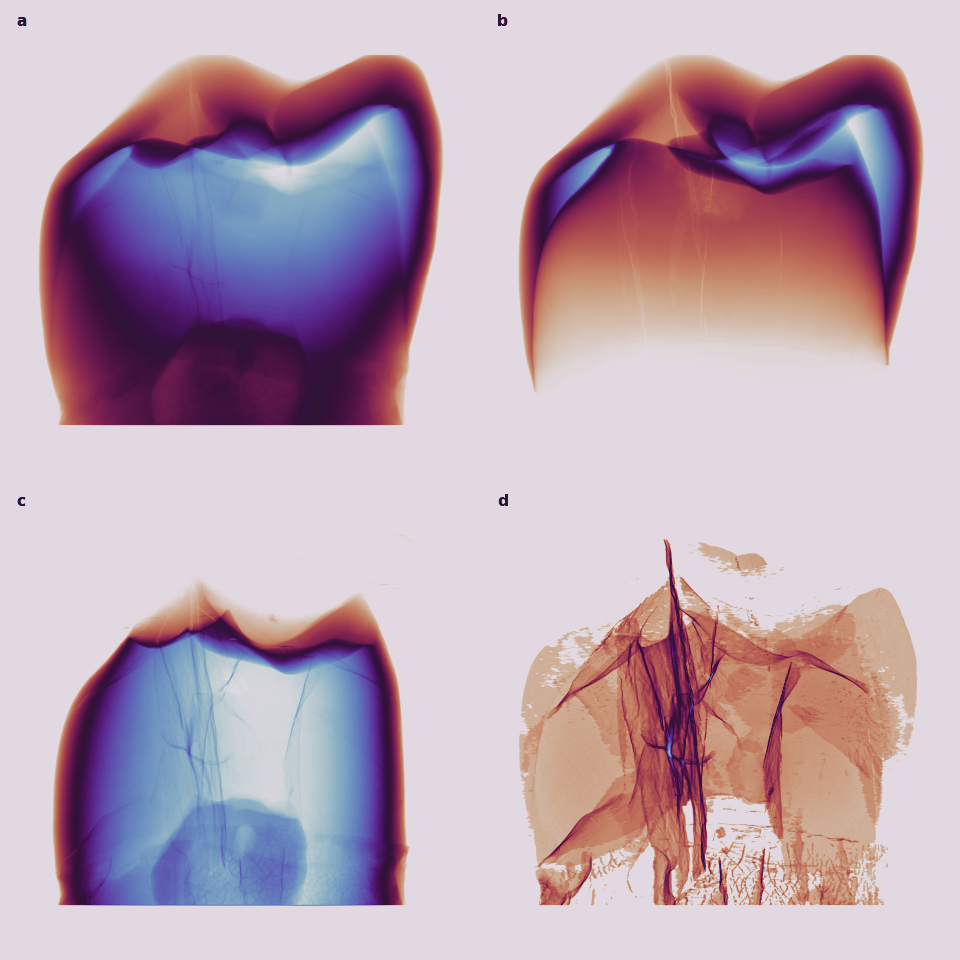

Supplement: Supplementary file 1 — Supplementary Information 1. [file 41598_2022_27062_MOESM1_ESM.gif]

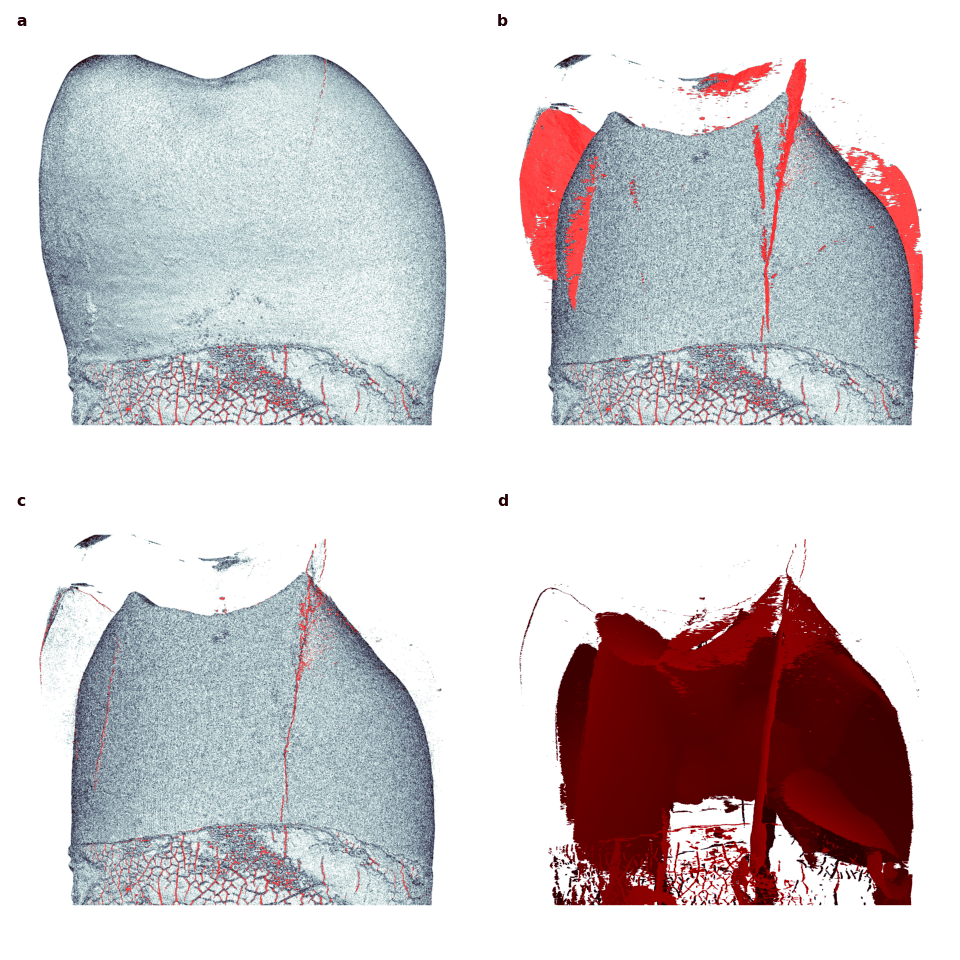

Supplement: Supplementary file 2 — Supplementary Information 2. [file 41598_2022_27062_MOESM2_ESM.gif]

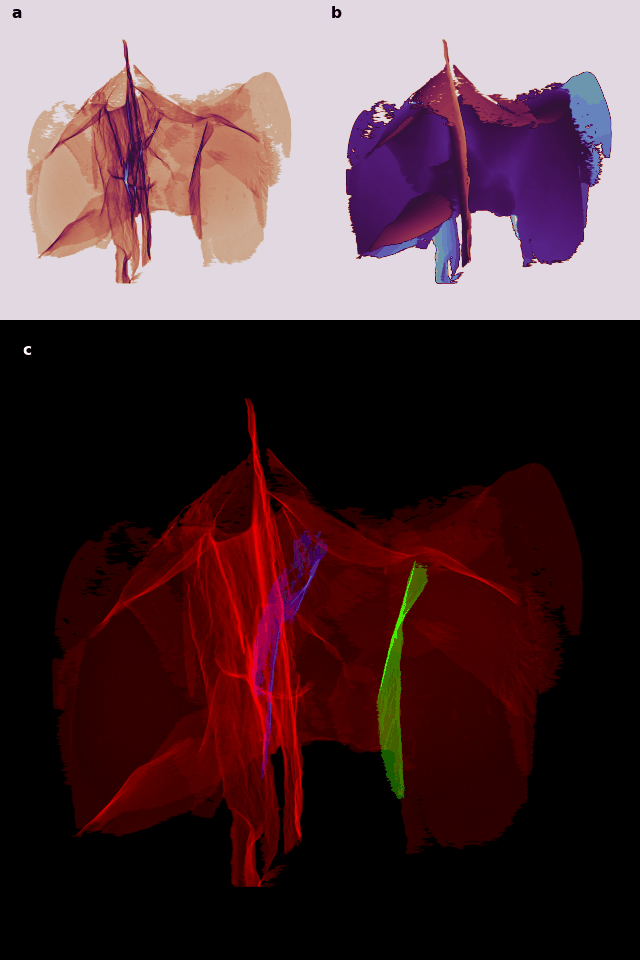

Supplement: Supplementary file 3 — Supplementary Information 3. [file 41598_2022_27062_MOESM3_ESM.gif]

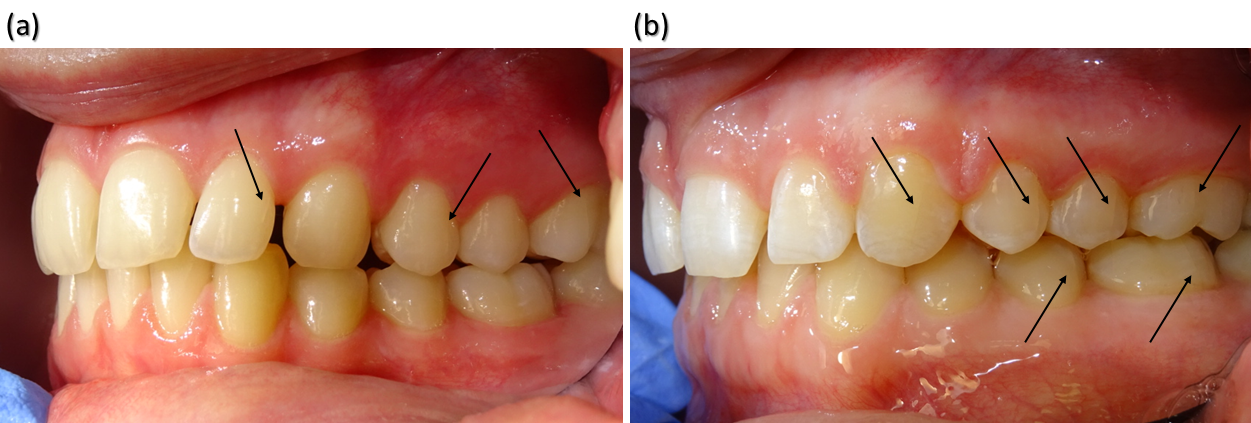

Supplement: Supplementary file 5 — Supplementary Information 5. [file 41598_2022_27062_MOESM5_ESM.png]
