# Supplementary material for: Revelation of microcracks as tooth structural element by X-ray tomography and machine learning
Source: Sci Rep. 2022 Dec 28;12:22489. doi: 10.1038/s41598-022-27062-5 (PMC9797571; doi:10.1038/s41598-022-27062-5)
Supplement: Supplementary file 6 — Supplementary Information 6. [file 41598_2022_27062_MOESM6_ESM.docx]

**Supplementary material for “Revelation of microcracks as tooth structural element by X-ray tomography and machine learning”**

**Fig. S1**. An example of photographs of (**a**) several teeth and (**b**) the whole group of posterior teeth with enamel microcracks on the buccal surface.

**Movie 1**. Same as Fig. 5. Panels show: **a** tooth, **b** enamel, **c** dentin, and **d** cracks. Data-cube is rotated with 1 deg steps. “NumPy 1.24.0”^49^ (https://numpy.org) was used to process data-cubes, which were visualized with “Matplotlib 3.6.0”^50^ (https://matplotlib.org).

**Movie 2**. Same as Fig. 6. Panels show: **a** enamel, **b** enamel cracks, **c** dentin, **d** dentin cracks. Data-cube is rotated with 1 deg steps. “NumPy 1.24.0”^49^ (https://numpy.org) was used to process data-cubes, which were visualized with “Matplotlib 3.6.0”^50^ (https://matplotlib.org).

**Movie 3**. Same as Fig. 7. Panels show: **a** projected density map (sum), **b** distance to the nearest crack, **c** three largest connected crack groups. Data-cube is rotated with 1 deg steps. “NumPy 1.24.0”^49^ (https://numpy.org) was used to process data-cubes, which were visualized with “Matplotlib 3.6.0”^50^ (https://matplotlib.org).

**Movie 4**. Same as Fig. 8. Panels show four teeth in our study sample. Data-cube is rotated with 1 deg steps. “NumPy 1.24.0”^49^ (https://numpy.org) was used to process data-cubes, which were visualized with “Matplotlib 3.6.0”^50^ (https://matplotlib.org).
